# Large-scale organoid study suggests effects of trisomy 21 on early fetal neurodevelopment are more subtle than variability between isogenic lines and experiments

**DOI:** 10.3389/fnins.2022.972201

**Published:** 2023-02-03

**Authors:** Jan T. Czerminski, Oliver D. King, Jeanne B. Lawrence

**Affiliations:** ^1^Medical Scientist Training Program, Department of Neurology, University of Massachusetts Chan Medical School, Worcester, MA, United States; ^2^Department of Neurology, University of Massachusetts Chan Medical School, Worcester, MA, United States; ^3^Department of Pediatrics, University of Massachusetts Chan Medical School, Worcester, MA, United States

**Keywords:** Down syndrome, neurodevelopment, iPS cells, cerebral organoids, Alzheimer’s disease

## Abstract

This study examines cortical organoids generated from a panel of isogenic trisomic and disomic iPSC lines (subclones) as a model of early fetal brain development in Down syndrome (DS). An initial experiment comparing organoids from one trisomic and one disomic line showed many genome-wide transcriptomic differences and modest differences in cell-type proportions, suggesting there may be a neurodevelopmental phenotype that is due to trisomy of chr21. To better control for multiple sources of variation, we undertook a highly robust study of ∼1,200 organoids using an expanded panel of six all-isogenic lines, three disomic, and three trisomic. The power of this experimental design was indicated by strong detection of the ∼1.5-fold difference in chr21 genes. However, the numerous expression differences in non-chr21 genes seen in the smaller experiment fell away, and the differences in cell-type representation between lines did not correlate with trisomy 21. Results suggest that the initial smaller experiment picked up differences between small organoid samples and individual isogenic lines, which “averaged out” in the larger panel of isogenic lines. Our results indicate that even when organoid and batch variability are better controlled for, variation between isogenic cell lines (even subclones) may obscure, or be conflated with, subtle neurodevelopmental phenotypes that may be present in ∼2nd trimester DS brain development. Interestingly, despite this variability between organoid batches and lines, and the “fetal stage” of these organoids, an increase in secreted Aβ40 peptide levels—an Alzheimer-related cellular phenotype—was more strongly associated with trisomy 21 status than were neurodevelopmental shifts in cell-type composition.

## Introduction

Cognitive disability is a universal feature of Down Syndrome (DS), and while the genetic basis of DS is clear—trisomy 21—how an extra copy of this tiny chromosome carrying ∼250 coding genes causes this phenotype is not well understood. Nor is it clear which neural cell-types, tissue and brain regions are impacted, or when impacts occur. Many studies have raised important hypotheses for specific cell phenotypes and mechanisms, but these various findings have not been consistently supported, and in some cases are conflicting. For example, several studies have suggested that interneuron number may be decreased in DS patients and human cell models (Ross et al., 1984; Bhattacharyya et al., 2009; Huo et al., 2018), in contrast to other reports in trisomic mice and human organoids that interneuron numbers are increased (Chakrabarti et al., 2010; Das et al., 2013; Xu et al., 2019).

In recent years, with the advent of high-throughput sequencing approaches, studies have begun to examine differences in the transcriptomes of DS versus euploid samples, but at this early stage there are few consistent conclusions. Studies almost invariably agree that many chr21 genes are upregulated in DS tissues and cells, although the number and identity of these genes varies between studies. For example, one meta-analysis of 45 transcriptome studies found 77 chr21 genes to be consistently upregulated in DS samples (Vilardell et al., 2011) and a more recent meta-analysis of 67 different studies including mouse and human datasets found 67 “consistently upregulated” genes on chr21 (De Toma et al., 2021). If there is no feedback regulation of a specific gene, a ∼1.5-fold increase in mRNA levels would be expected, although sensitivity to detect this relatively modest change will depend on the power of the experimental design. Numerous sources of variability between samples (e.g., genetic background, cell-type proportions in sample, pathological state, age, sex, etc.) may especially weaken the power to detect differences in non-chr21 genes and pathways impacted by chr21 dosage. Thus, it remains a challenge to identify consistent changes directly (or indirectly) due to trisomy 21, or, as considered here, avoid conflating differences due to other biological variables with differences caused by trisomy. Table 1 summarizes several sources of variation that may complicate interpretation of iPSC disease modeling and indicates the strategies used in this study to minimize each of these.

**TABLE 1:** Potential sources of variability in iPSC disease modeling.

Sources of variability	Strategies used in this study to lessen variability
Genetic differences between individuals	All-isogenic cell lines
Differences in isogenic clones from different reprograming events	Subclones from same reprograming event
Differences between “identical” sub-clones A. Evolution during culture Genetic drift Epigenetic drift B. Freeze/thaw bottleneck Genetic drift Epigenetic drift	Expand lines to six (three disomic and three trisomic)
Differences between individual organoids	Pooling large numbers of organoids
Differences between differentiations	Four repetitions using a semi-directed (relatively consistent) protocol

Most recently, several studies have reported that trisomy 21 causes broad transcriptome-wide changes, with some studies reporting global genomic dysregulation (Mowery et al., 2018) or the presence of domains of up- and down-regulation across the genome (Letourneau et al., 2014). However, the latter phenomenon has been called into question (Do et al., 2015), was not seen in other recent studies (Gonzales et al., 2018; Moon and Lawrence, 2022), and was seen in both normal and trisomic samples (Ahlfors et al., 2019). In comparison of normal and DS brain tissue hundreds or more non-chr21 genes have been found differentially expressed under stringent statistical cut-offs (e.g., Lockstone et al., 2007); however, such findings can reflect differences in cell-type proportions in tissue samples, age, or pathological states. For example, some evidence from post-mortem DS brain samples and human cellular models find increased numbers of astroglia (Mito and Becker, 1993; Lu et al., 2011; Zdaniuk et al., 2011; Briggs et al., 2013; Chen et al., 2014). Such differences in cell-type proportions or tissue status alone could account for broad transcriptome changes in brain samples, complicating identification of specific pathways directly perturbed by trisomy 21.

Numerous molecular pathways and specific chr21 genes (for example, *DYRK1A*, *RCAN1*, *OLIG2*, and *OLIG1*, *DSCAM*, *SOD1*, *PCNT* and several IFN-receptor genes) are hypothesized to be central to DS, although consensus has not been reached. Modestly smaller fetal brain sizes have been reported in DS fetuses; however, DS infants have developmental milestones closer to normal at birth and adults often score as more severely impacted than children. Hence, it is important to better understand when in human pre-natal and/or post-natal periods the cognitive deficits arise. This is key to therapeutic strategies, and has been studied primarily in DS mouse models (Ruparelia et al., 2012; Bartesaghi et al., 2015). Recently, new non-invasive *in utero* imaging technologies make studies of human brain development more feasible; for instance, a recent study of DS fetuses by MRI (Patkee et al., 2020) suggests some reduction in cerebellar volume in late second trimester compared to controls. While expanding such studies will be important, methods are needed to investigate and better define the cellular neurodevelopmental changes, including cell-types, functions and molecular pathways impacted—information essential for the development of effective therapeutic targets and strategies.

Recently, methods to generate cerebral organoids from human pluripotent stem cells have emerged as a new model system for early human neurodevelopment, modeling 3D brain tissues (Di Lullo and Kriegstein, 2017). Organoid systems model development of more complex tissues with a variety of cell-types, and over a longer time frame than standard 2D neural cultures. Protocols for specific brain regions continue to be developed, in what is a promising but young and rapidly evolving research approach.

This study began with the goal of using organoid technology to model the impact of trisomy 21 on human fetal neurodevelopment. Our unexpected results raise significant questions regarding the extent of early fetal neurodevelopmental changes due to trisomy 21. The progression of results throughout this work is also instructive for the use and interpretation of neurodevelopmental modeling using stem cells. As our efforts evolved, we progressively developed improved experimental design strategies, which we believe are informative more broadly for organoid and stem cell approaches to study neurodevelopmental conditions.

Our lab’s recent DS studies used an inducible XIST transgenic system to directly compare the same trisomic cell line with and without “trisomy 21 silencing” (Chiang et al., 2018; Czerminski and Lawrence, 2020; Moon and Lawrence, 2022); however, to study 3D cortical organoids here, we employed the more common approach of comparing isogenic trisomic and disomic cell lines. Comparison of isogenic cell lines avoids differences in genetic background in the cells of origin, although isogenic human iPSC lines can evolve epigenetic differences, as will be discussed. After assessing three organoid generation protocols, we chose to utilize a directed forebrain spheroid method to compare organoids generated from isogenic trisomic and disomic cell lines. Having found cytological markers for cell-types difficult to quantify in a sufficiently large number of organoids, we conducted in-depth transcriptomics to identify differentially expressed genes and to computationally deconvolve cell-type proportions in organoids formed from three trisomic and three disomic isogenic cell lines.

The focus here is on investigating neurodevelopmental deficits in this model of early fetal brain development, but we also briefly examined a neurodegenerative-related pathology linked to triplication of the chr21 *APP* gene and early onset Alzheimer Disease (AD). Some individuals with DS develop amyloid plaques as early as adolescence, and ∼80% show clinical dementia by ∼60–65 years (Wiseman et al., 2015). The *APP* gene is clearly a driver of AD in DS (Wiseman et al., 2015; Hithersay et al., 2019), causing increased production of Aβ peptides by cleavage of APP (Lehmann et al., 2018; Fortea et al., 2020). While plaques generally develop over time, an increase in soluble Aβ may be present very early and has been reported in trisomic organoids (Gonzalez et al., 2018; Alic et al., 2021). Hence, we determined whether an increase in soluble Aβ was detected in our system, for comparison and perspective in relation to our neurodevelopmental findings, and as an indicator of the sensitivity of the experimental design.

Results presented here make methodological points that have value for the field of disease modeling with human iPSCs more generally, but at the same time the specific results have significant implications for understanding the developmental biology of trisomy 21.

## Materials and methods

### iPSC culture

The isogenic cell lines used here were generated and characterized as described in our prior study (Jiang et al., 2013), and expanded to identify six all-isogenic subclones, derived from the same DS iPSC parental line (DS1-iPS4) (Park et al., 2008). In characterizing ∼100 subclones for the prior study (focused on creating *XIST* transgenic lines), we identified many subclones that were not transgenic for *XIST* (but had the tet-puromycin selection gene). Some subclones were shown to be euploid by spontaneous loss of one chromosome 21, with chr21 transcriptome levels equivalent to non-isogenic normal control cells (Jiang et al., 2013). Several such trisomic and disomic subclones were isolated, expanded and preserved for future studies and used as controls cells (lacking XIST) in various contexts (Chiang et al., 2018; Czerminski and Lawrence, 2020; Moon and Lawrence, 2022). iPSCs were maintained on vitronectin-coated plates with Essential 8 medium (ThermoFisher, Waltham, MA, USA) and tested periodically for mycoplasma. Cells were passaged every 3–4 days with 0.5 mM EDTA. Cell lines were verified for appropriate chromosome 21 number by FISH for a chr21 gene (e.g., *APP*) before each series of differentiations, and trisomy 21 status confirmed by RNA sequencing transcriptomics.

### Cerebral organoid generation

As described in the supplement, we first briefly compared three organoid generation protocols (Supplementary Figure 1 legend), and for consistency and ease of use we chose for RNA-seq studies the forebrain spheroid protocol previously described (Pasca et al., 2015), with significant alterations. iPSCs were re-aggregated in 96-well plates in iPSC media containing 20 ng/ml thermostable FGF-2 (Millipore, Burlington, MA, USA) and 50 μM Y-27,632 (Tocris Bioscience, Minneapolis, MN, USA). The next day, half the media was exchanged with neural differentiation media (NDM) containing 2 μM DMH1 (Tocris Bioscience, Minneapolis, MN, USA) and SB431542 (Tocris Bioscience, Minneapolis, MN, USA). Organoids in individual wells were fed with this media every day for 14 days. After 14 days, media was changed to neural media containing 20 ng/ml FGF-2 and EGF (Peprotech, Cranbury, NJ, USA) as described (Pasca et al., 2015) and moved to ultra-low attachment 24-well plates (Corning, Tewksbury, MA, USA). From this point forward, organoids were grown on an orbital shaker set at ∼100 RPM to improve aeration. At day 32, FGF-2 and EGF were replaced with 20 ng/ml of BDNF (Peprotech, Cranbury, NJ, USA) and NT-3 (Peprotech, Cranbury, NJ, USA) for 18 days. At day 50, organoids were fed every other day with neural media without any supplements.

### Cell fixation, RNA FISH, and immunofluorescence

Forebrain organoids were fixed for 30 min in PFA at room temperature, washed three times with PBS, and cryopreserved in 30% sucrose/PBS at 4°C overnight. Fixed organoids were embedded in O.C.T. compound (Sakura Finetek, Torrance, CA, USA), frozen in an isopropanol/dry ice slurry, and sectioned at 14 μm on a cryotome. Sections were attached to Superfrost Plus slides (Electron Microscopy Sciences, Hatfield, PA, USA) and stored at −20°C until staining. Prior to staining, sections were rehydrated in PBS for 5 min, and detergent extracted in 0.5% Triton X-100 (Roche, Indianapolis, IN, USA) for 3 min.

Immunofluorescence was performed as previously described (Clemson et al., 1996; Byron et al., 2013). Fixation with 4% paraformaldehyde was performed prior to detergent extraction. The primary antibodies used in this study are provided in Table 2. The conjugated secondary antibodies used in this study were Alexa Fluor 488, 594, and 647 (ThermoFisher, Waltham, MA, USA).

**TABLE 2:** Primary antibodies.

Antibody	Host	Source	Identifier
NeuN	Mouse monoclonal	Millipore, Burlington, MA, USA	MAB377
Sox2	Rabbit polyclonal	Millipore, Burlington, MA, USA	AB5603
TUBB3 (Tuj1)	Mouse monoclonal	Biolegend, San Diego, CA, USA	MMS-435P
Sox1	Goat polyclonal	R&D systems, Minneapolis, MN, USA	AF3369
GFAP	Rabbit polyclonal	MilliporeSigma, Burlington, MA, USA	AB5804
PAX6	Rabbit polyclonal	Biolegend, San Diego, CA, USA	901,301

### RNA isolation, cDNA library preparation, and high-throughput sequencing

Whole organoids were washed once with 1X PBS and placed into 2 ml microcentrifuge tubes containing one 5 mm steel bead (Qiagen, Venlo, Netherlands) and 1 ml of Trizol reagent (ThermoFisher, Waltham, MA, USA). These samples were homogenized using the TissueLyser II instrument (Qiagen, Venlo, Netherlands) on the P1 setting. Beads were then removed using a magnet and samples were either stored at −80°C or RNA extraction, DNAse treatment, and RNA cleanup was performed immediately.

RNA was extracted using TRIzol reagent (ThermoFisher, Waltham, MA, USA) according to manufacturer’s instructions. RNA samples were cleared of contaminating genomic DNA by DNAse I (Roche, Indianapolis, IN, USA) treatment for 1 h at 37°C. RNA cleanup and DNAse I removal was performed using RNeasy MinElute columns (Qiagen, Venlo, Netherlands) according to manufacturer’s instructions. Clean RNA was assessed for quality on an Advanced Analytical Fragment Analyzer. All samples had an *RQN* > 7.5 and strand-specific sequencing libraries were prepared using the NEBNext Ultra II Directional RNA Library Prep Kit for Illumina in conjunction with the NEBNext Poly(A) mRNA Magnetic Isolation Module and NEBNext Multiplex Oligos for Illumina (New England Biolabs, Ipswich, MA, USA).

Sequencing was performed by the UMass Chan Medical School Deep Sequencing Core Facility on the Illumina HiSeq 4000 platform to a depth of ∼8 million reads/sample (2 × 50bp paired-end) in the case of the large organoid experiment or on the NextSeq 500 instrument to a depth of ∼30 million reads/sample (2 × 38bp paired-end) in the case of the pilot experiment.

### RNA sequencing analysis

Reads were aligned to the GRCh37/h19 human genome build using hisat2 (Kim et al., 2019) (v2.0.5) with Ensembl gene annotations (release 87) (with added entry for the Tet/Puro transgene). Strand-specific read counts for each gene were computed using the featureCounts function of the subread package (Liao et al., 2019) (v1.6.2). The R package edgeR was used for library normalization (TMM method) and differential expression testing, using quasi-likelihood tests (prior.count = 2, robust = T) that account for uncertainty in the estimates of dispersions to give more rigorous control over false positives (Lun et al., 2016). In the large organoid experiment, replicate samples and repeated differentiations of the same cell line were summed together to form a 3 vs. 3 comparison, to avoid false positives due to pseudoreplication (Lazic, 2010). Following edgeR recommendation (Lun et al., 2016), genes with very low expression were filtered out prior to analysis (right before the library normalization step); only genes with counts per million (CPM) of at least 0.25 in at least half the samples were retained, a CPM that corresponds to roughly 8 reads in the pilot study and 11 reads in the large organoids study (after summing replicates).

The false-discovery rate (FDR) (Benjamini and Hochberg, 1995) was used to control for multiple comparisons. Because chr21 consists of a small number of genes (∼1% of genome), a strong signal that is specific to chr21 can be diluted in this genome-wide FDR due to many thousands of non-chr21 genes with large *p*-values. (Conversely, a strong signal that is specific to chr21 can result in some non-chr21 genes being declared significant under the FDR just by virtue of being lumped in with the chr21 genes.) Therefore, to better isolate the evidence for differential expression of genes on or off chr21—a distinction that is *a priori* relevant for DS studies—FDRs are computed separately for chr21 and for non-chr21 genes, denoted by FDR_*chr*21_ and FDR_*non*21_, respectively. Since many studies do not calculate FDR for chr21 and non-chr21 separately, we also report the usual genome-wide FDR in supplemental tables and illustrate the *p*-value cut-off for *FDR* < 0.1, along with *FDR*_*chr*21_ < 0.1 and *FDR*_*non*21_ < 0.1, on the volcano plots in Figures 2B, 6A, B. (Note: The *p*-value cut-offs are the largest *p*-values for which the corresponding *FDR* < 0.1; when none of the *N* off-chr21 genes satisfied *FDR*_*non*21_ < 0.1 the *p*-value cut-off was indicated as 0.1/*N*, the Bonferroni cut-off—this is more stringent than the FDR cut-off in general, but if a hypothetical single gene were to satisfy the FDR cut-off it would also satisfy the Bonferroni cut-off).

Cell-type deconvolution was performed using the DWLS-WLS (dampened weighted least-squares) method in the R package DLWS (Tsoucas et al., 2019) based on the 13 reference cell-types defined in the integrative analysis of Tanaka (Tanaka et al., 2020). Deconvolution was done on a linear scale, with bulk RNA-seq counts normalized as FPKM (fragments per kilobase of exon per million mapped fragments) to adjust for the gene-length bias in bulk RNA-seq, and single-cell reference profiles normalized as CPM (counts per million), which were divided by 100 to avoid sporadic numerical convergence issues with DWLS-WLS. Reference profiles for each cell-type were computed separately for cells from the Birey (Birey et al., 2017) and Quadrato (Quadrato et al., 2017) datasets included in the Tanaka study, using count data and cell-type assignments from https://data.mendeley.com/datasets/3wrtkk4w5v/2. The Birey dataset uses the Pasca protocol, which corresponds more closely to the protocol used here than the modified Lancaster protocol used for the Quadrato dataset, but this had fewer than 10 cells assigned to several of the reference cell-types. Because of this, the average of the Birey and Quadrato basis vectors was used for deconvolution in the main results reported here. Marker genes for each of the 24 clusters from the Tanaka study were downloaded from https://cells.ucsc.edu/?ds=organoidatlas and ranked by *p*-value with ties broken by fold-change. For cell-types corresponding to single clusters, the top 30 marker genes were used for the basis vectors. For cell-types comprising more than one cluster, the top five marker genes from each cluster were selected and the remainder of the 30 genes were selected based on rank in combined marker lists for these clusters. Ribosomal and mitochondrial genes (bsecsc function getMITRIB) (Baron et al., 2016) were excluded from the basis vectors, and chr21 genes were also excluded to avoid any direct effect of chr21 gene dosage on estimated cell-type proportions.

For tests of differential expression with cell-type representation adjustment, the estimated proportion of cortical neurons in the summed replicates was included as an additive covariate in the edgeR statistical model. (A covariate for only one of the cell-types was included to avoid depleting the residual degrees of freedom, and cortical neurons had the largest interquartile range in estimated proportion across samples).

For tests of differences in cell-type proportions associated with trisomy 21, Welch’s *t*-tests were used—these do not assume equal variance between groups so can better accommodate the reduction in variance that may happen for proportions near zero. In the large organoid study, estimated proportions were based on the summed replicates for each line, for a 3 vs. 3 comparison. Bonferroni correction was used to control for testing for differences in multiple cell-types.

The R package ggplot2 (Wickham, 2016) was used to generate most graphs.

### Aβ Analysis

48-h old media was removed from tissue culture wells containing individual organoids. Media was immediately placed on ice and centrifuged at 2,000 rcf for 5 min to remove cell debris. Media supernatant was stored at −80°C. ELISA was performed using the ultrasensitive Amyloid beta ELISA kit from Invitrogen (ThermoFisher, Waltham, MA, USA) (Paina et al., 2011) per manufacturer’s instructions with media samples diluted 1:2 in standard diluent buffer. Only Aβ40 was measured in this study, since the Aβ42 ELISA kit was unavailable at the time, and focused analyses of AD pathologies are the subject of another study. Plates were read at 450 nm using a BioTek EL800 microplate reader (Agilent Technologies, Santa Clara, CA, USA).

The R package drm (Ritz et al., 2015) was used to fit a 4-parameter (LL.4 model) standard curve to ODs in a dilution series with seven concentrations, and this was used to convert sample ODs to estimated Aβ concentrations. Because several trisomic samples had ODs higher than any of the calibration samples, their estimated concentrations depended strongly on the upper asymptote of the standard curve, which could not be confidently estimated from the calibration data. To avoid unstably inflated estimates of concentrations for ODs that are close to this asymptote, we clamped the parameter “Upper” in the LL.4 model to the value 3.0 while fitting the standard curve, which gives conservative extrapolations. The reported results change only slightly when clamping this parameter to other values above 2.4, so are not sensitive to the precise choice of 3.0.

Differences in Aβ concentration will be impacted by organoid cell-counts, which we could not directly measure. So, we used RNA concentration as a proxy for cell-count and included it as an additive covariate in a linear mixed-effect model along with fixed effects for trisomy state and batch and random effects for cell line and its interaction with batch [lmer function in the R package lme4 (Bates et al., 2015)]. *P*-values and confidence intervals were computed with the contest function in the R package lmerTest (Kuznetsova et al., 2017) using Satterthwaite’s method. Both the RNA and Aβ concentration were log2 transformed in this model, with estimated effect sizes and confidence intervals then converted back to linear fold-changes. The normalized Aβ concentrations shown in Figure 7 were obtained by subtracting off the estimated effect of RNA concentration, then converting back to a linear scale. Note that the estimated effect of RNA concentration on Aβ concentration was small (coef = 0.087) and non-significant (*p* = 0.71), and similar results were obtained by averaging un-normalized log2 Aβ concentrations for each cell-line and performing a *t*-test on these 3 vs. 3 averages. This use of RNA levels as a proxy for organoid size/cell-count improves reliability of the comparisons in what are nonetheless approximate Aβ levels in this analysis.

## Results

The results presented detail the progression of experimental design improvements based on results of our initial observations. The last and largest experiment was formulated from lessons learned from our initial cerebral organoid studies, which highlighted the need to address several sources of variation that are often present in iPSC modeling, but not due to trisomy 21. DS studies have reported numerous phenotypic and transcriptional differences attributed to trisomy in human or mouse neural tissues and cells; however, it is often difficult to know whether other potential differences between samples have been ruled out. In our initial experiments, comparing individual isogenic organoids from one trisomic and one disomic line, we found intriguing differences. Given this, we then sought to determine whether these differences might be accounted for by variation (unrelated to trisomy 21) between organoids, experiments, or cell lines—even isogenic cell lines. Realizing the need to expand the experimental design led us to focus on quantitative transcriptome analyses comparing hundreds of organoids from experimental repetitions, and, importantly, generated from additional all-isogenic iPSC lines. While the need for large numbers of organoids to control for variation between organoids was not unanticipated, our results indicate that the number of isogenic iPSC lines needed to confidently investigate a potentially subtle neurodevelopmental phenotype was more than initially expected.

### Generation of organoids using a directed forebrain spheroid protocol

**FIGURE 1:**
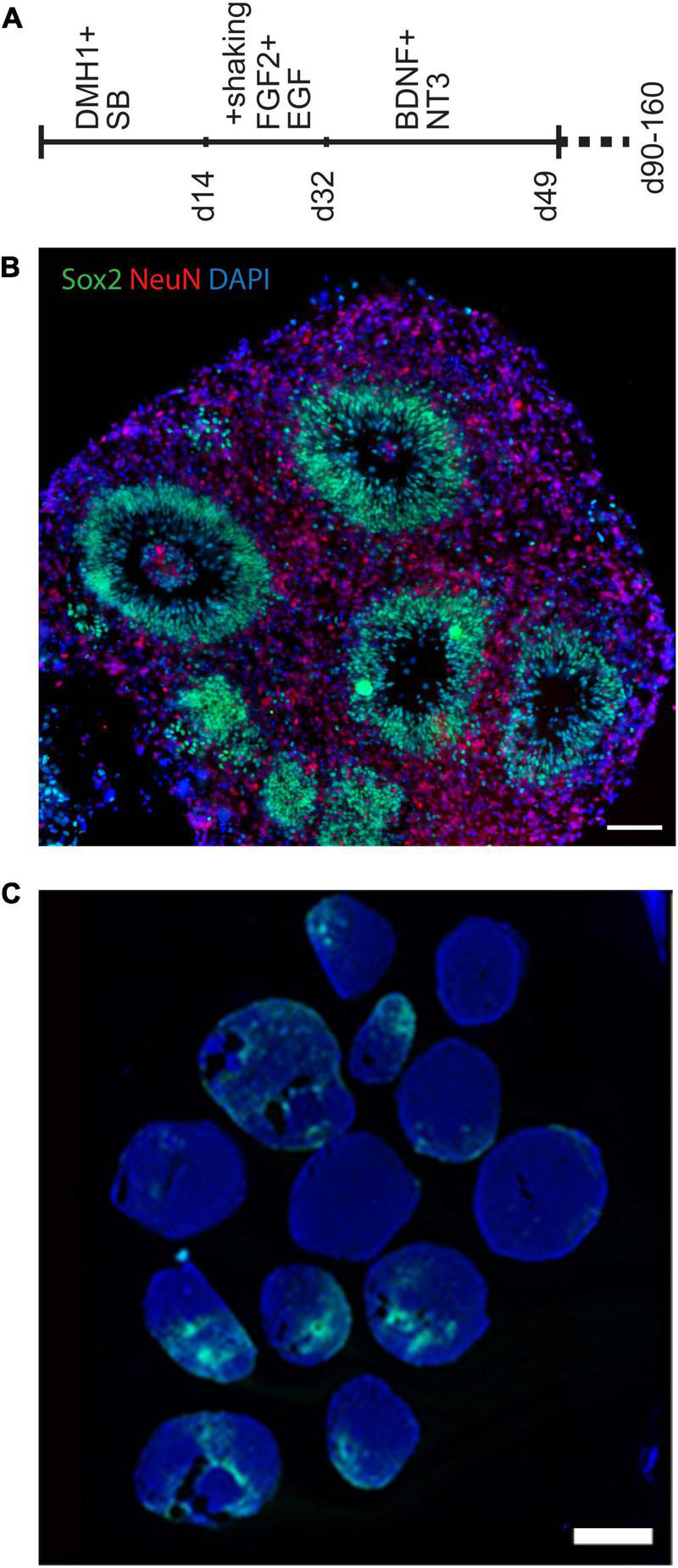
Generation of organoids using a directed forebrain spheroid protocol. **(A)** Visual summary of modified Pasca protocol used to generate organoids. **(B,C)** Immunofluorescence photomicrographs. **(B)** This modified protocol produced large spheroids containing smaller VZ-like zones along with some unorganized progenitor-containing areas. **(C)** Prolonged culture with this protocol generated significant numbers of GFAP-expressing cells, which include astrocytes. Wide variability between individual organoids can be seen, which makes quantification of cell representation difficult. Scale bars are 100 μm in panel **(B)** and 1 mm in panel **(C)**.

Several 3D cell culture models of cerebral development have recently been developed, each with its own set of advantages and drawbacks. We first created organoids using three different protocols (Lancaster et al., 2013; Lancaster and Knoblich, 2014; Pasca et al., 2015; Qian et al., 2016) to identify one which we found most tractable and reproducible in our hands, as summarized in the supplement. Protocols that use minimal exogenous patterning molecules can be advantageous for certain purposes, but can produce structures representing very different brain regions that vary between organoids. In order to reduce this aspect of variability and compare organoids modeling a more defined brain region, we decided to use the “directed” protocol described by Pasca (Pasca et al., 2015) to generate forebrain spheroids, which we modified slightly to make more tractable in our hands. A visual summary of this organoid differentiation protocol is provided in Figure 1A, and further described in the supplement. The Pasca protocol utilizes dual-SMAD inhibition, high concentrations of the mitogens FGF2 and EGF, as well as the neurotrophins BDNF and NT3 to generate spheroids that include only cortical-like cells, including both neurons and astroglia. After ∼50 days, these organoids formed a large number of well-organized ventricular-like zones (VZs) (Figure 1B) containing neural progenitor cells surrounded by a neuron-containing cortical plate region.

We had initially planned to examine differences between organoids using cytological staining for specific cell-type markers. For example, prolonged culture with this protocol generates significant numbers of GFAP-expressing cells, which include astrocytes (as well as radial glial progenitors) (Figure 1C and Supplementary Figure 1E). The figures illustrate the variability of GFAP staining seen between numerous organoids within each pool, providing the first indication that large numbers of organoids would need to be examined to control for this variability, and large-scale quantification of such a cytological assay is challenging. Therefore, after early attempts to analyze potential differences in cell-type representation using histological methods, we came to the conclusion that variability from organoid to organoid (even in a less variable directed protocol) made accurate quantification particularly difficult (Figure 1C and Supplementary Figure 1E). For this reason, we turned to bulk RNA sequencing, from which cell-type representation in trisomic and disomic organoids can be estimated using deconvolution algorithms, based on reference expression profiles of the constituent cell-types.

### Small comparison of isogenic organoids from a trisomic and a disomic line show differences that might be due to trisomy

Our first pilot RNA sequencing experiment used bulk RNA-seq of 10 organoids aged for 160 days, five from a trisomic (parental) line and five from an isogenic euploid control line. Isogenic lines have the same genetic background, and comparison of subclones derived from the same iPS parental line avoids differences in reprograming or somatic cell of origin. The overall strategy was to generate bulk sequence data and use published gene sets and expression profiles for different cell-types to deconvolve the cell-type representation in each sample. In this first experiment we chose to evaluate the variation between individual organoids by sequencing the 10 organoids individually.

**FIGURE 2:**
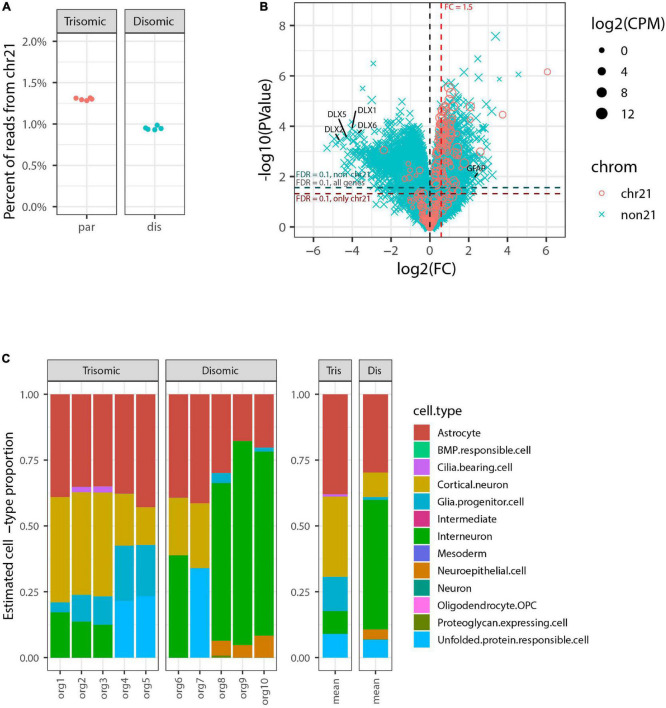
Genome-wide transcriptome analysis of pilot organoid experiment. **(A)** Percent of total RNA-seq reads that are from genes on chromosome 21 in each individual organoid sequenced (5 trisomic and 5 disomic). Trisomic organoids have close to the expected 1.5-fold increase in overall chromosome 21 expression compared to disomic organoids. **(B)** Volcano plot for tests of differential expression between trisomic and disomic organoids. Chr21 genes are represented by magenta circles, other genes by cyan x symbols; size indicates average expression level of gene. Dashed red vertical line indicates 1.5-fold elevation in trisomic organoids, and dashed horizontal lines represent *p*-value cut-offs corresponding to *FDR* = 0.1 for all genes, and also separately for chr21 genes and non-chr21 genes. DLX family genes and *GFAP* are labeled. **(C)** Estimated cell-type composition of each organoid based on deconvolution with respect to reference profiles derived from published single-cell RNA-seq data (see Methods for details). The barplot to the right shows averages of these estimates for the five trisomic and five disomic organoids.

We generated RNA-seq data for each of the 10 organoids to a depth of ∼30 million reads and then compared RNA-seq data for the five trisomic and five disomic organoids. This data showed a clear difference in overall expression of genes from chr21 between individual organoids from the trisomic versus the disomic iPSC lines (Figure 2A). In tests for differential gene expression between the trisomic and disomic samples (Figure 2B) we observed extensive differences both for genes on chr21 (cyan) and not on chr21 (magenta), with 105 chr21 and 5,662 non-chr21 differentially expressed genes (DEGs) at *FDR* < 0.1 (out of 229 chr21 and non-chr21 20,567 genes total, after filtering out genes with very low expression as detailed in Methods). As explained in the methods, to avoid diluting the signal for differential expression among the small proportion of genes on chr21, we also computed FDRs separately for chr21 genes (designated FDR_*chr*21_) and non-chr21 genes (FDR_*non*21_). Here because there were so many DEGs off of chr21 this had only a modest effect, with 111 chr21 genes having *FDR*_*chr*21_ < 0.1 and 5,626 non-chr21 genes having *FDR*_*non*21_ < 0.1. These 111 chr21 genes included 10 with higher expression in disomic than trisomic samples, which may reflect differences between particular cell-lines or organoid-batches. The DEGs include genes with various non-coding biotypes and genes with lowish average expression, which contribute to the high number of non-chr21 DEGs, but 78% (4,399) were for protein-coding genes, 87% (4,917) for genes with average CPM > 1, and 75% (4,207) for both. As discussed in the introduction, differences of several hundred up to a few thousand non-chromosome 21 genes are a common finding in published studies of DS.

In Figure 2B we have highlighted that the non-chr21 DEGs included many DLX family genes, all of which were downregulated on average in the five trisomic relative to the five disomic organoids. These genes are well-known for involvement in the specification and migration of ventral forebrain-derived interneurons (Anderson et al., 1997; Stuhmer et al., 2002; Cobos et al., 2007; Paina et al., 2011), and there have been mixed reports in human samples and cell models of whether interneuron generation is decreased or increased due to trisomy (Ross et al., 1984; Bhattacharyya et al., 2009; Huo et al., 2018; Xu et al., 2019).

To further investigate whether the overall expression pattern of interneuron-related marker genes was indicative of altered interneuron representation, and to examine representations of other cell-types, we used computational deconvolution to estimate cell-type composition of each sample based on markers genes and reference expression profiles derived from a published integrative analysis of single-cell RNA-seq datasets from brain organoids (Tanaka et al., 2020) (see Methods for details). Tests of differences in estimated cell-type composition between trisomic and disomic organoids indicated nominally significant differences (*p* < 0.05 by Welch’s *t*-test before multiple hypotheses correction) for three cell-types—glial progenitor cells, cortical neurons, and interneuron—but after Bonferroni-correction these were no longer statistically significant, with *p* ≥ 0.23 for all cell-types (see Supplementary Table 1).

Importantly, in this pilot study we sequenced the ten total organoids each individually, for perspective on the extent of individual organoid variability, which would inform how many organoids should be studied. This revealed that differences in DLX genes seen in the average were driven largely by a subset of disomic organoids whereas some disomic organoids had similar interneuron composition to the trisomic organoids. Similarly, glial progenitor cells were overrepresented on average in the trisomic organoids, and there was an increase in expression of *GFAP* (a gene also expressed in astrocytes) (Figure 2B). However, the data for individual organoids shows the proportion has high variability between individual organoids, and is not well correlated with trisomy 21. These results illustrate that despite using a directed organoid generation protocol, significant variability between individual organoids in interneuron formation weakens any conclusions that can be drawn on this point.

Even if consistent significant differences had been seen between organoids generated from these two cell isogenic cell lines, a key question would remain as to whether that difference is due to the presence of trisomy 21, or to other sources of variation between samples, including between isogenic cell lines cultured separately (Table 1). Since human pluripotent cells are epigenetically fragile and sensitive to culture or freeze/thaw conditions, differences in cell populations commonly evolve. For example, our lab and others previously showed that different lab isolates of the same hESC line, or even colonies within the same culture, often show epigenetic differences (e.g., in chromosome regulation, XIST RNA expression, chromatin marks, differentiation, and nuclear structures) (Hall et al., 2008; Lund et al., 2012; Halliwell et al., 2020).

### Expanded experimental design indicates cell-type representation differences do not correlate with trisomy 21

The above findings provide suggestive differences in organoid development from the two cell lines, although these were only nominally significant. A more powerful experimental design could affirm or discount that these differences are consistently correlated with trisomy 21. The above results indicated that many more organoids should be examined for each line, but we also recognized that differences observed between any two particular lines could reflect differences unrelated to trisomy 21. Thus, the experimental design was greatly expanded to include many more organoids, more isogenic lines, and more experimental repetitions (Table 3), in order to increase the power to discriminate differences due to trisomy from differences due to other factors. A total of over 1,100 organoids (Table 3 and Figure 1A) from three trisomic and three disomic all-isogenic iPSC lines (see Methods) were generated. To minimize effects of individual organoid differences, we examined pools of 12 organoids, four pools per each of six cell lines, and repeated this scheme in four independent batches of organoids. Roughly half of these organoids were used for bulk RNA sequencing, with separate RNA-seq libraries constructed for each of two pools for each cell line in each of four experimental repetitions, for a total of 48 RNA-seq samples: eight samples for each of six lines (Figure 1B). The remaining organoids were frozen for histology and media preserved to assay for Aβ secretion, and for other future analyses on parallel samples.

**TABLE 3:** Expanded organoid experiment.

• Three disomic subclone lines	• Three trisomic subclone lines
• Pools of 12 organoids per sample	• Pools of 12 organoids per sample
• Quadruplicate samples per expt = 144	• Quadruplicate samples per expt = 144
• Repeat full expt 4 times	• Repeat full expt 4 times
= 576 Disomic organoids	= 576 Trisomic organoids

Minimize sources of variation using six lines and >1,100 isogenic organoids.

**FIGURE 3:**
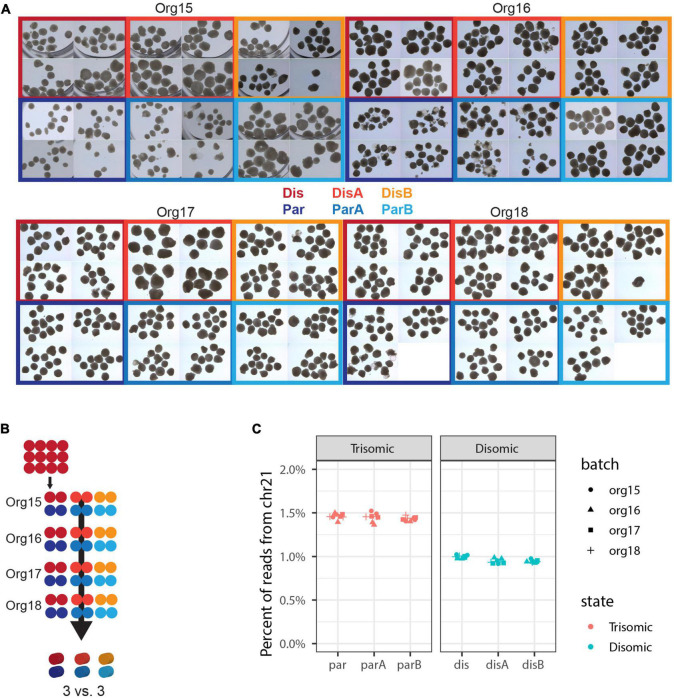
Expanded experimental design to discriminate differences due to trisomy 21. **(A)** Micrographs of nearly all organoids generated in this experiment. Independent differentiations are signified by “org”, isogenic trisomic lines by “par”, and isogenic disomic lines by “dis”. **(B)** Schematic of samples generated. Each of the 48 dots represents 12 organoids and one sample for sequencing, while the 3D cylinders signify *in silico* collapsing for statistical comparisons. **(C)** Percent of total RNA-seq reads that are from genes in chromosome 21 in each of the 48 pools of 12 organoids.

Initial sequencing analysis confirmed the expected ∼1.5 fold higher overall chromosome 21 expression in all trisomic versus all disomic lines (Figure 3C).

**FIGURE 4:**
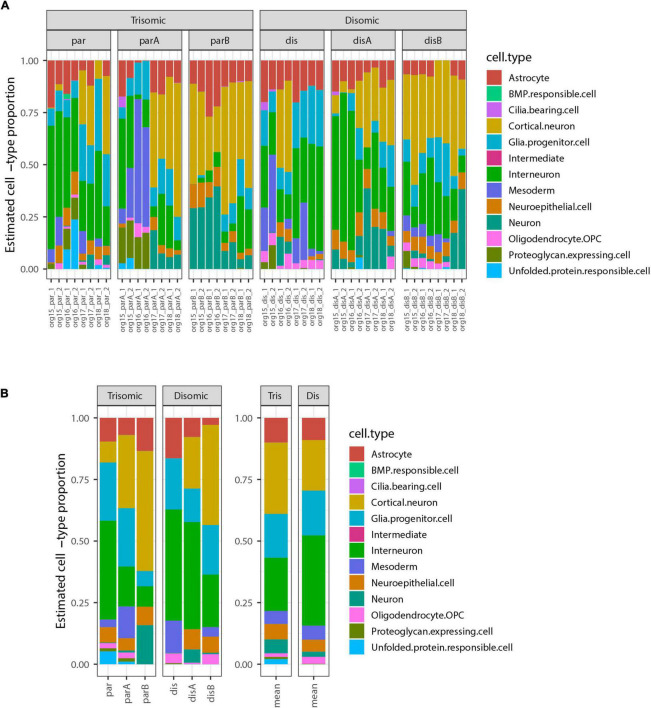
Estimated cell-type composition in expanded organoid experiment design to discriminate differences due to trisomy 21. **(A)** Estimated cell-type composition for each of 48 pools of 12 organoids based on deconvolution with respect to reference profiles derived from published single-cell RNA-seq data (see Methods for details). **(B)** Estimated cell-type composition for each cell-line, based on collapsing RNA-seq data from 8 organoid pools for that cell-type prior to deconvolution (used for statistical analyses). The barplot to the right shows the averages of these estimates for the 3 trisomic and 3 disomic cell lines.

We next used the bulk transcriptome data to estimate the cell-type composition, as in the pilot study. The most highly represented cell-types were cortical neurons and interneurons, which form neighboring clusters in the UMAP projection in the study from which these cell-types were defined (Tanaka et al., 2020). There were also notable contributions from astrocytes, glial progenitor cells, and neuroepithelial cells. Surprisingly, this analysis revealed that some organoid samples contained a subset of mesoderm-derived cells (Figure 4), suggesting some degree of off-target differentiation.

Notably, there were no statistically significant differences in the estimated proportions of the reference cell-types between the disomic and trisomic states, with all *p*-values > 0.2 before multiple hypothesis correction and all Bonferroni-corrected *p*-values equal to 1 (Figure 4 and Table 4; Supplementary Table 2). Differences in cell-type representations between cell lines of the same state (disomic or trisomic) were apparent, but differences were not consistent between the disomic versus trisomic lines.

**TABLE 4:** Differences in estimated cell-type proportions in disomic and trisomic samples from the expanded organoid study shown in Figure 4.

Cell-type	Ave disomic	Ave trisomic	Difference	*P*-value	*P*-adj. Bonf.
Astrocyte	9.01e–02	1.00e–01	−9.96e–03	0.836	1
BMP responsible cell [sic]	0.00e + 00	2.42e–19	−2.42e–19	0.423	1
Cilia bearing cell	2.27e–06	3.64e–21	2.27e–06	0.423	1
Cortical neuron	2.05e–01	2.90e–01	−8.46e–02	0.636	1
Glia progenitor cell	1.82e–01	1.79e–01	3.33e–03	0.962	1
Intermediate	7.10e–18	3.95e–18	3.15e–18	0.638	1
Interneuron	3.66e–01	2.15e–01	1.51e–01	0.290	1
Mesoderm	5.66e–02	5.32e–02	3.46e–03	0.953	1
Neuroepithelial cell	4.90e–02	6.25e–02	−1.35e–02	0.646	1
Neuron	2.08e–02	5.63e–02	−3.54e–02	0.567	1
Oligodendrocyte OPC	2.86e–02	1.48e–02	1.39e–02	0.378	1
Proteoglycan expressing cell	1.35e–03	8.75e–03	−7.40e–03	0.229	1
Unfolded protein responsible cell [sic]	1.93e–21	2.08e–02	−2.08e–02	0.322	1

Columns are as follows: Cell-type: Cell-types from Tanaka et al., 2020 used as basis for deconvolution of bulk RNA-seq data; Ave disomic: Mean of estimated proportion of cell-type in disomic samples; Ave trisomic: Mean of estimated proportion of cell-type in trisomic samples; Difference: Difference between these means (ave disomic—ave trisomic); *P*-value: *p*-value for Welch *t*-test; *P*-adj Bonf: Bonferroni-corrected *p*-value, to control for multiple hypothesis testing (one test per cell-type) (names are taken directly from metadata at https://cells.ucsc.edu/?ds=organoidatlas&meta=Dataset, and there are two cell types for which the word “responsible” was used instead of “response”).

Comparison of results for a given line between independent differentiations suggested that some variation appears sporadic but some may reflect inherent epigenetic differences between even isogenic cell lines, which may evolve in culture (see Table 1). We cannot rule out that the variability between cell lines may mask the possible presence of more subtle differences in the propensity of disomic and trisomic organoids to form different neural cell-types. Also, cell-type proportions estimated by deconvolution are known to depend on many factors including choice of reference cell-types, selection of marker genes, the algorithm used, and data normalization steps (Avila Cobos et al., 2020). We explored several options for these factors and sometimes observed substantial changes in estimated proportions, so these should not be regarded as definitive; however, the lack of significant differences in estimated proportions between disomic and trisomic lines was a consistent finding.

### Analysis of organoid size highlights variability independent of trisomy 21

**FIGURE 5:**
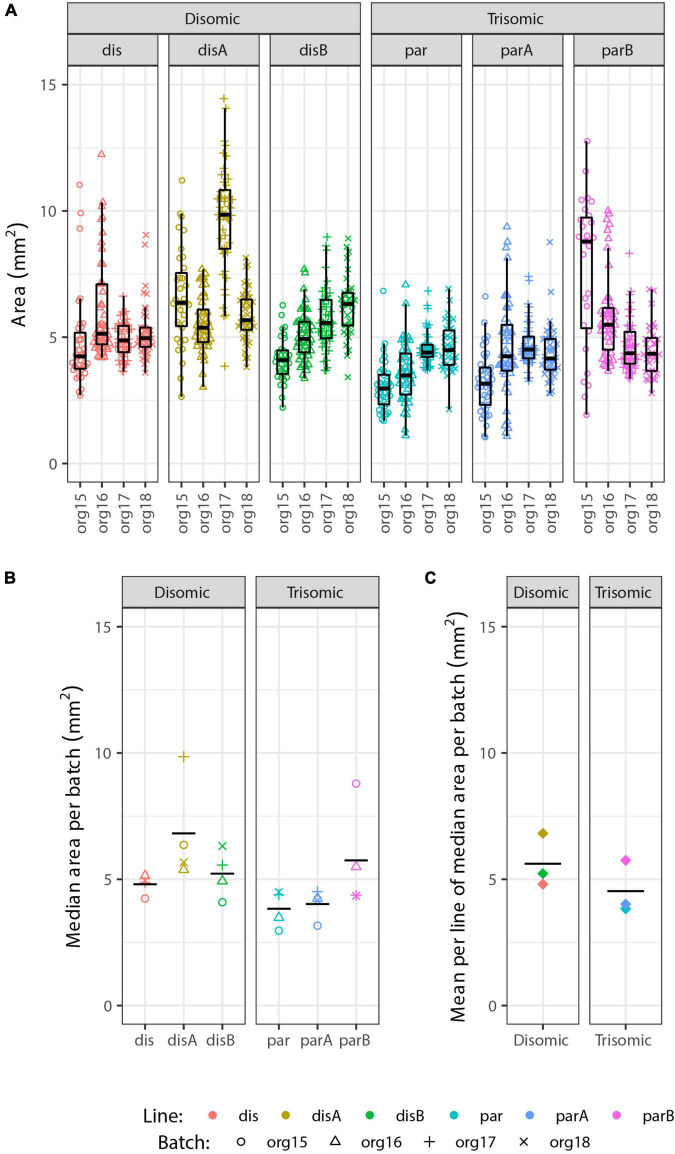
Analysis of organoid sizes in expanded organoid study. Measurements of areas of 90-day organoids from 3 disomic and 3 trisomic subclone lines, from 4 experiments (org15, org16, org17, and org18) for each, each consisting of ∼40 organoids per line (see Figure 3A for micrographs of organoids). There are clear differences in average organoid sizes for particular lines in particular experiments **(A)**, and a suggestion of differences between lines in the average sizes across all experiments **(B)**, but the overall difference in average size between disomic and trisomic lines is not significant here **(C)** (*p* = 0.27 by Welch *t*-test). Statistical power is limited by variance between lines and the relatively small number of lines (3 vs. 3).

Irrespective of the cell-type composition, we also considered whether differences in organoid size may be correlated with trisomy 21. The presence of the extra chromosome may confer increased cell stress (Oromendia et al., 2012; Sheltzer et al., 2012; Bonney et al., 2015) or cell senescence (Nawa et al., 2019; Meharena et al., 2022), which could reduce general cell proliferation. Hence, we also examined whether the overall size of trisomic organoids was smaller than disomic organoids, as seen in a recent study (Tang et al., 2021). Each of 93 pools of approximately twelve 90-day organoids was photographed (as illustrated in Figure 3A) and the diameter of each organoid measured, and results are summarized in Figure 5. The graphs provide useful perspective on the extent of variability in individual organoids of the same sample, replicate organoid batches for specific cell-lines (Figure 5A), and differences between specific cell-lines averaged across experiments (Figure 5B). These levels of variation need to be accounted for in assessing whether the smaller overall average for the trisomic versus disomic lines (Figure 5C) can be concluded to be due to trisomy 21. The difference is not near statistical significance (*p* = 0.28 by 3 vs. 3 Welch *t*-test on mean per line of median organoid size in each batch). Even with several hundred organoids compared in four replicate experiments, the statistical power is limited by the number of isogenic trisomic and disomic lines. Multiple organoids from the same “batch” or replicate batches (experiments) from the same cell line are not independent samples for comparing the effects of trisomy 21, because results will be impacted by the particular size propensity of particular cell lines (or batches) compared.

We emphasize that these findings do not discount that there could be a mild growth disadvantage conferred by trisomy 21, even potentially in organoids, but results here highlight that the inter-cell-line (and inter-batch) variability can be a complicating factor in such studies. In fact, we found decreased proliferation of undifferentiated trisomic iPS cells in a prior study that directly compared cultures of the same cell line with and without induced silencing of one chr21 (Jiang et al., 2013). Comparison of human fibroblast lines (non-isogenic) suggested the trisomic lines examined were more susceptible to replicative senescence in culture (Swanson, 2014) (dissertation chapter IV), consistent with other recent evidence that trisomy 21 can increase cell senescence under stress (Oromendia et al., 2012; Marcovecchio et al., 2021). Hence, we do rule out there is some impact of trisomy 21 muted in our study, but we note that expression of p16 and p21 (senescence markers) were not increased in trisomic organoids, consistent with the statistically nonsignificant differences in organoid size.

### Strong detection of chr21 gene upregulation contrasts with paucity of genome-wide DEGs

A theme of recent studies in DS cells and tissues is the finding of extensive transcriptome-wide differences between trisomic and euploid samples. This also might be suggested in our initial comparison of small numbers of organoids from a single trisomic iPSC line and a single disomic subclone. However, the analysis of the six isogenic iPSC line panel found that individual lines differed in the cell-type proportions they tended to produce, and these differences did not correlate with trisomy 21. Since deconvolution infers cell-type proportions from expression levels of cell-type marker genes, comparisons between lines or organoid samples that differ in cell-type composition will have statistically significant DEGs that just reflect cell-type differences, irrespective of trisomy 21 status. If not properly accounted for in the statistical analysis, multiple replicates or multiple differentiations from the same cell-line can amplify cell-line-specific differences resulting in small *p*-values that reflect real biological differences between lines or samples, but, importantly, these are not necessarily due to trisomy 21. This is a form of pseudoreplication (Hurlbert, 1984), in which small *p*-values may only provide evidence of a difference between the *particular samples* studied. Thus, in our RNA-seq analysis we first collapsed read-counts for replicate samples and samples from different differentiations of each cell line—in effect averaging together expression of the ∼100 organoids generated in each cell line—prior to performing a comparison of the three trisomic vs. three disomic lines (Figure 3B).

**FIGURE 6:**
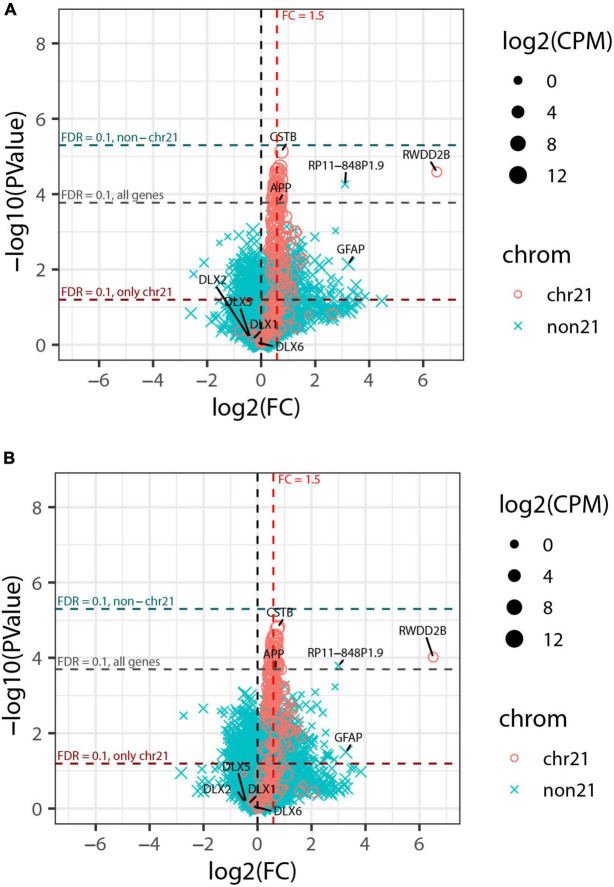
Genome-wide transcriptome analysis of expanded organoid experiment. **(A)** Volcano plot of collapsed 3 vs. 3 comparison of trisomic and disomic lines. Chr21 genes are represented by magenta circles, other genes by cyan x symbols; size indicates average expression level of gene. Dashed red vertical line indicates 1.5-fold elevation in trisomic lines, and dashed horizontal lines represent *p*-value cut-offs corresponding to *FDR* = 0.1 for all genes, and also separately or chr21 genes and non-chr21 genes. **(B)** Volcano plot of the same comparison as in panel **(A)** but including the estimated percent of cortical neurons as a covariate for each sample. The labeled genes are: selected genes on chr21 (*CSTB*, *APP*, and *RWDD2B*); the non-chr21 gene with the smallest *p*-value (*RP11-848P1.9*); *GFAP* and DLX-family genes that were differentially expressed in the pilot study but did not meet FDR cut-offs for significance here.

This analysis detected strong upregulation of genes across chromosome 21, with 74% (120 or 163) of genes expressed at >1 CPM differentially expressed at *FDR*_*chr*21_ < 0.1, as were 65% (139 of 214) of all analyzed chr21 genes. Most chr21 DEGs were at or near the theoretical 1.5-fold higher expression expected in trisomic cells (Figure 6A); this is illustrated by *CSTB* and *APP*, which are highly expressed, in contrast to a notably large fold-change for *RWDD2B*, which is more lowly expressed. The most striking finding, however, was that despite robust detection of chromosome 21 DEGs, only a single non-chr21 gene was differentially expressed at *FDR* < 0.1 in this greatly expanded experiment—the pseudogene *RP11-848P1.9* on chr17—and none when the FDR was computed separately for just non-chr21 genes (FDR_*non*21_). Neither *GFAP* nor the DLX genes noted in the smaller pilot study were near the cut-off for significance in the expanded study (Figure 6A).

This indicates that the extensive genome-wide differences found in the pilot study (Figure 2B) reflect differences between the small organoid samples from the particular cell lines rather than differences that can be attributed to trisomy *per se*. Given the paucity of non-chr21 DEGs in this expanded study, we also considered that inconsistent cell-type representations between the various cell lines/samples will increase expression variability that could weaken detection of gene expression changes between trisomy and disomy groups. To address this, we tested differential expression using a model that accounts for the estimated proportion of cortical neurons in each sample, as this was the cell-type with highest variability between samples (based on variance and IQR). As seen in Figure 6B, this adjustment had only a modest effect on the overall results, increasing chr21 genes with *FDR*_*chr*21_ < 0.1 from 139 to 143, and genes off of chr21 with *FDR*_*non*21_ < 0.1 remaining at zero (and just one at *FDR* < 0.1). Results from tests of differential expression without and with adjustment for cortical neuron proportions are provided in Supplementary Table 3 (which also includes results for pilot study).

The strongest conclusion from these results is that the presence of widespread differences in expression in the one-line vs. one-line comparison is not validated with a stronger experimental design. Other biological variation, such as in cell-type representation, may be associated with hundreds or more non-chr21 DEGs, not necessarily a direct or indirect effect of trisomy 21. While this overall finding is clear, whether any individual gene is identified as a DEG can be modestly impacted by even small differences in estimated cell-type proportions and specific cut-offs. Thus, changes in the deconvolution methodology, filtering of lowly expressed genes and other computational details can affect whether a few off-chr21 genes are identified as significant, as in our earlier analysis of this data (Czerminski, 2019; Czerminski et al., 2022). Hence, our central conclusion here is that this expanded organoid study shows powerful detection of chr21 DEGs, but that we cannot affirm that chromosome 21 dosage broadly impacts the transcriptome in this model of fetal brain development.

### Over-production of Aβ is evident in fetal-stage trisomic organoids

**FIGURE 7:**
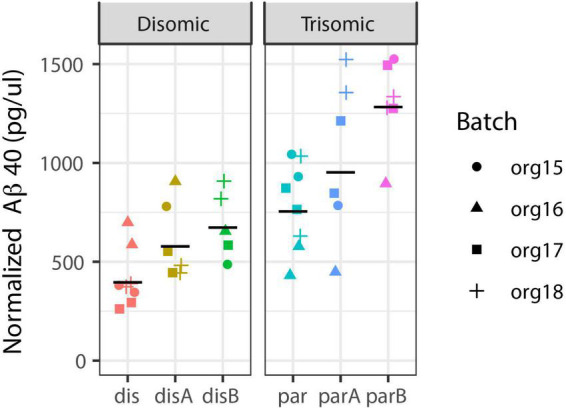
Aβ40 secretion is increased in trisomic organoids. Aβ40 levels were measured in media from pools of disomic **(left)** and trisomic **(right)** organoids: Two pools from each of four batches from six cell-lines (three trisomic and three disomic, all-isogenic). Estimated increase is 1.73-fold, with 95% CI from 0.94-fold to 3.18-fold (mixed effect model with Satterthwaite’s method; see Methods for details). Black line = geometric mean of all replicates for cell-line.

While an in-depth study of AD-related cell phenotypes is the subject of a separate study, we include here a limited analysis of *A*β to contrast detection of this neurodegenerative pathology to the neurodevelopmental results. We isolated media from each organoid pool and analyzed whether an increase in secreted Aβ would be detected in these fetal stage organoids, using ELISA for Aβ40 (see Methods). Figure 7 shows data for two replicates of four independent differentiations (organoid batches) from the three trisomic and three disomic isogenic iPSC lines. Note that there is variability between isogenic trisomic lines, and between the disomic lines, and some variation between experimental batches. Such variation might be expected given the differences in cell-type representation in these samples, since certain cell-types (e.g., neurons) express more APP and Aβ. Interestingly, even against the backdrop of this variability, and without correction for cell-type composition in samples, soluble Aβ40 levels—related to a neurodegenerative phenotype (Alzheimer’s Disease)—showed a stronger association with trisomy 21 (*p* = 0.068; see Methods) than differences related to neurodevelopment (as reflected by cell-type proportions; *p* > 0.2 for all cell-types even without multiple-hypotheses correction).

## Discussion

This study began with the expectation that isogenic comparisons of trisomic and disomic cortical organoids, as models of early fetal neurodevelopment, would reveal differences in cell-types and pathways caused by trisomy 21. Understanding how and when brain development and/or function is impacted in DS is critical to understanding the biology of trisomy 21 and to assess therapeutic prospects to mitigate cognitive or neurological deficits in DS (Hasina et al., 2022). Given the nature of our results, this study evolved to focus substantially on methodological considerations, providing significant technological insights for disease modeling with iPSCs and organoids. However, the methodological considerations should not overshadow the significant biological implications of our “negative findings”, which bear on the extent to which trisomy 21’s impacts on neurodevelopment are manifest in fetal cortical development, at least as reflected in this 90-day organoid model. Overall findings indicate that, in this model (of ∼early 2nd trimester development), any effects of trisomy 21 on specific cell-types (or the genome-wide transcriptome) are sufficiently subtle to be obscured by the biological/technical variance of our organoid system. While we began with the presumption that substantial differences are present (and will be detected), in the end the overall findings challenge our presumption and raise the important question of how much trisomy 21 has indeed impacted neurodevelopment by this approximate early stage. Hence, to avoid selective publication bias, we felt the this “finding” (lack of detectable neurodevelopmental changes in the large-scale organoid study) should be published, as it has potential implications for the developmental biology of DS, and for this research field. Many more studies will be required to resolve these questions, some of which may require more studies using non-invasive *in utero* imaging in large numbers of developing fetuses.

Numerous studies report a variety of differences comparing trisomic and euploid samples, including in various mouse models, different human DS tissues, and various cell-based models. Inconsistencies in findings between studies may be due to differences in the systems examined, or in some cases limitations in sample size, etc. Human iPSC studies have the potential to provide more controlled comparisons between trisomic and euploid cells/organoids in comparable developmental/functional states. However, iPSC cultures are prone to environmental changes that can affect experimental results (Klein et al., 2022) and organoids can exhibit substantial between-organoid differences but also batch-to-batch variability in cell composition (Hernandez et al., 2022). Hence adequately controlling for several levels of technical/biological variation in the labor-intensive culture and differentiation of stem cells is a challenge. Inter-organoid variability is more widely recognized, but results here highlight that comparisons are also impacted by common uncontrolled differences (epigenetic or genetic) that often evolve during the separate culture of isogenic human pluripotent cell clones and sub-clones [for example (Hall et al., 2008; Lund et al., 2012; Halliwell et al., 2020)]. We worked to minimize as practical several sources of variation that complicate disease modeling (summarized in Table 1), and suggest consideration of these levels of variation should guide experimental design and statistical analysis, to avoid pseudoreplication of sample-specific differences that may be conflated with effects of trisomy.

From the start we used a totally isogenic system, using isolated lines (subclones) derived from a single reprograming event, avoiding the differences between isogenic lines from distinct reprograming events or cell of origin effects, which can manifest as differences in neural differentiation potential (Koyanagi-Aoi et al., 2013). Because we used multiple large organoid pools per sample in repeated experiments, we believe we adequately controlled for organoid variability in the large-scale study. Large numbers of pooled organoids were generated from each of six isogenic lines (three trisomic and three disomic) and the entire large organoid production scheme was repeated four times, allowing us to account for variability between organoid experiments (batch effects).

With these efforts, we improved detection of the relatively subtle 1.5-fold increase in expression of individual chromosome 21 genes, with ∼75% of chr21 genes expressed at >1 CPM differentially expressed due to trisomy (*FDR*_*chr*21_ < 0.1). This was more than the 58% of chr21 genes satisfying the same criteria in the smaller (pilot) experiment, and more chr21 DEGs than reported in many studies of DS tissues or cells, which, paradoxically—and as in our pilot study—report many more off-chr21 DEGs than found in the expanded experiment here (Vilardell et al., 2011; Weick et al., 2013; Letourneau et al., 2014; Olmos-Serrano et al., 2016; Mowery et al., 2018). Despite strong detection of chr21 DEGs in the more powerfully designed experiment, there were few if any off-chr21 DEGs, dramatically fewer than the ∼5,000 DEGs in the pilot experiment. The smaller experiment comparing five organoids each from a trisomic and a disomic line detected broad genome-wide expression differences that are statistically significant and may reflect “real” differences (between properties of particular cell lines or small organoid samples or batches), but this does not mean they are due to trisomy 21. Results of the expanded study indicate these sources of variation need to be accounted for before one can confidently conclude differences between samples are caused by trisomy 21.

Consistent with a lack of abundant non-chr21 DEGs, the more powerful experimental design did not detect statistically significant differences in cell-type representations linked with trisomy 21 status. Despite examining large numbers of organoids for each line, we still saw considerable variability between cell lines of the same chr21 state. There was relatively good consistency between duplicate samples (pools of 12) for each organoid experiment (Org 15, 16, 17, 18 in Figure 4A), suggesting this number of organoids provides a reasonably representative sample. Four repetitions of the organoid experiments helped mitigate what we found is significant variation between organoid experiments of the same line, although just a few “batches” out of 24 showed especially marked differences. Perhaps most importantly, individual cell lines tended to generate cell-type representations that were consistent across organoid experiments, but differed between isogenic cell lines (of the same status with respect to trisomy 21). Thus, when all eight pooled samples for the three disomic and the three trisomic lines are compared (Figure 4B), we did not validate our initial expectation and found that differences in cell-type proportions did not correlate with trisomy 21.

We emphasize that we do not conclude from this that there are no differences present in this fetal-stage of cortical development, or even in this forebrain organoid model, but that if trisomy 21 specific neurodevelopmental effects are present, they are too limited to rise above the experimental “noise” in our organoid study. Even with experimental noise, stronger effects can still be more detectable in organoids, as illustrated by our results for secreted Abeta, which, ironically, is linked to development of amyloid plaques and neurodegeneration at later ages. Our findings are not directly comparable to other studies due to significant differences in the organoid generation protocols or other parameters. We used organoids patterned toward a specific forebrain subregion as in Xu (Xu et al., 2019) in order to decrease some of the variability in organoids. This has some advantages, but could miss differences in specific cell-types that are not well-represented, such as oligodendrocytes or potentially glial cells. Thus, our findings do not contradict these studies, but highlight the challenge that discerning neurodevelopmental differences appears sensitive to experimental design. Previous studies in iPSC-derived DS cells have described a range of findings with many reporting no difference in the neuronal differentiation capacity of DS cells (Shi et al., 2012; Briggs et al., 2013; Lu et al., 2013; Weick et al., 2013; Gonzales et al., 2018). Other studies using unrelated disomic and trisomic iPSCs in a monolayer culture system have demonstrated an increase in the proportion of astroglia formed by trisomic cells (Chen et al., 2014). Another recent study generated patterned ventral forebrain organoids using DS cells and found an increase in the propensity of trisomic cells to form interneurons, which was correctible by knockdown of a chr21 gene, *OLIG2* (Xu et al., 2019). This finding contrasts with previous studies in iPSCs and primary human cells that describe the opposite finding (Ross et al., 1984; Bhattacharyya et al., 2009; Huo et al., 2018).

Overall, results of our study raise caution about false-positive results, but also potential false negative results, in assessing neurodevelopmental phenotypes that may be quite subtle and difficult to model with iPSCs, as discussed elsewhere (Soldner and Jaenisch, 2012). Studying large numbers of organoids per subclone provides part of the remedy, but this is insufficient; the power to detect differences related to trisomy can still be limited by the number of isogenic lines studied, since differences between even subclones maintained as separate lines can over-shadow milder phenotypes (or be conflated with trisomy phenotypes). Epigenetic instability in human pluripotent cell lines, as reflected in changes in chromatin modifications or *XIST* RNA regulation, have been seen to evolve even between individual colonies within the same cell culture dish (Hall et al., 2008; Silva et al., 2008), hence we cannot rule out that this is epigenetic/genetic drift that can arise during long organoid culture, or the experimental repetitions. To circumvent potential effects of inter-cell line differences, we are currently testing an inducible *XIST*-based system which compares organoids of the same trisomic line with and without silencing of trisomy 21 (Czerminski and Lawrence, 2020). While this may circumvent this part of the challenge, development of protocols that produce more uniform organoids will also be important to realize the full promise of organoid modeling for Down syndrome, or other neurodevelopmental syndromes.

## Data availability statement

The data presented in this study are deposited in the NCBI Gene Expression Omnibus (GEO) repository, accession number GSE222365.

## Author contributions

JC and JL conceived and designed the study. JC performed the experiments. JC, OK, and JL analyzed the data and wrote the manuscript. All authors contributed to the article and approved the submitted version.
