# Supplementary material for: Large-scale organoid study suggests effects of trisomy 21 on early fetal neurodevelopment are more subtle than variability between isogenic lines and experiments
Source: Front Neurosci. 2023 Feb 3;16:972201. doi: 10.3389/fnins.2022.972201 (PMC9935940; doi:10.3389/fnins.2022.972201)
Supplement: Supplementary file 1 [file Presentation_1.pdf]

Supplementary Material

Evaluating three approaches for generating cerebral organoids with DS iPSCs

Since the choice of an organoid protocol is an important aspect of any study using this approach, prior to the in-depth RNAseq studies we generated and briefly compared organoids from three recently described protocols. We briefly describe this comparison to explain our choice of protocols for the main study, for which our priority was a protocol that modeled cortical neurogenesis and was reproducible and tractable in our hands. Each of the 3D cell culture models we tested has its own set of advantages and drawbacks; most significantly, protocols differ in the usage of exogenous patterning molecules. The Lancaster protocol (Lancaster et al.,2013; Lancaster and Knoblich,2014) uses minimal patterning and makes use of the "default fate" of differentiating pluripotent cells to become rostral neuroectoderm. Other protocols utilize patterning molecules to establish region-specific 3D models of human neurodevelopment. In particular, the Pasca protocol (Pasca et al.,2015) utilizes dual-SMAD inhibition, high concentrations of the mitogens FGF2 and EGF, as well as the neurotrophins BDNF and NT3 to generate spheroids that include only cortical-like cells, including both neurons and astroglia. A third protocol utilizes SMAD inhibitors as well as mild WNT signaling activation, which ameliorated apoptotic cell death and potentially further dorsalized the organoids (Qian et al.,2016; Qian et al.,2018).

A visual summary of the organoid differentiation protocols tested in this study is provided in Figure S1D. We compared these three protocols to get an overall impression of the consistency of results and ease of work-flow, using isogenic iPSCs with trisomy 21 and their disomic controls. The unpatterned cerebral organoids, as previously described (Lancaster et al.,2013; Lancaster and Knoblich,2014) (see Figure S1 legend), generated large ventricular-like zones with tightly packed neural stem cells surrounded by outwardly migrating postmitotic neurons (Figure S1A). However, these structures constituted a minority of cells in each organoid, which were largely composed of self-organizing cells in a non-ventricular pattern, reminiscent of choroid plexus-like tissue, as well as other cells lacking clear organization (Figure S1A'). Because our aim was to examine the effects of trisomy on cortical neurogenesis, we decided the variability in cerebral structures present from organoid to organoid made this this protocol insufficiently reproducible for our purposes.

Next, we created forebrain organoids based on (Qian et al.,2018), with minor modifications (see Figure S1 legend). After ~50 days, these organoids formed a large number of large, well-organized ventricular-like zones (VZs) (Figure S1B), and these organoids demonstrated a particularly striking contrast between neural progenitor cell-containing VZs and surrounding neuron-containing regions. This protocol may be favorable for certain purposes, but in our initial tests some batches did not produce the characteristic VZ-containing morphology (potentially due to inconsistencies in Matrigel embedding and subsequent disembedding (Figure S1B'). In addition, the increased labor required to generate organoids with Matrigel embedding, made this protocol more impractical to generate organoids with higher throughput.

To this end, we generated cortical spheroids based on (Pasca et al.,2015), with some significant modifications in order to consistently generate organoids. Most significantly, the exposure to SMAD inhibitors was increased from one to two weeks, and the subsequent mitogen and neurotrophin treatments were thus delayed by one week (see Fig 1A and Fig. S1D). This protocol produced large spheroids containing multiple VZ-like zones, and some unorganized progenitor-containing areas, and these were surrounded largely by neurons (Figure 1B and S1C). The somewhat smaller VZ zones were generally present, and this protocol also produced significant numbers of astrocytes and glia.

After early attempts to analyze potential differences in cell-type representation using histological methods, we came to the conclusion that the still-large degree of variability from organoid to organoid and cell line to cell line makes accurate quantification a particularly difficult task (Figure S1E).

Figure S1. Evaluating three approaches for generating cerebral organoids with DS iPSCs. A-C) Immunofluorescence photomicrographs of representative cortical regions in three selected organoid generation protocols. A') Non-cortical region in organoid generated with Lancaster protocol, which resembles choroid plexus in organization. B') Cortical region of organoid generated with Qian protocol but lacking the distinct radial organization seen in (B). D) Visual summary of protocol generation protocols. All protocols utilize the same first step of single-cell dissociation and re-aggregation in 96-well plates. E) 90-day organoids from each of the 6 cell lines (3 trisomic, top row; 3 disomic, bottom row), generated with protocol (see methods) and demonstrating robust GFAP expression, suggesting formation of astrocytes and/or radial glia. Wide variability between lines as well as variability between individual organoids from the same line can be seen. Scale bars are 100μm in (A-C) and 1mm in (E).

Lancaster protocol: organoids were generated as previously described (Lancaster et al.,2013; Lancaster and Knoblich,2014). Briefly, iPSCs were dissociated into single cells and plated at a density of ~9,000 cells/well in 96-well round-bottom ultra-low attachment plates (Corning) in iPSC media containing 4ng/ml thermostable FGF-2 (Millipore) and 50μM Y-27632 (Tocris Bioscience). After 6 days, organoids were transferred to ultra-low-attachment 24-well plates in N2 and heparin-containing neural induction media. Organoids were embedded in Matrigel droplets on day 11 of differentiation and grown for 4 days before transferring to an orbital shaker set at ~100 RPM.

Qian protocol: forebrain organoids were generated as previously described (Qian et al.,2016) with the following modifications: embryoid bodies were formed by dissociating iPSCs into single cells and re-aggregating in U-bottom 96-well plates (Lancaster and Knoblich,2014). On day 7, aggregates were transferred to ultra-low attachment 6-well plates (Corning) for Matrigel embedding, and on day 14 the plates were moved to an orbital shaker set at ~100rpm.

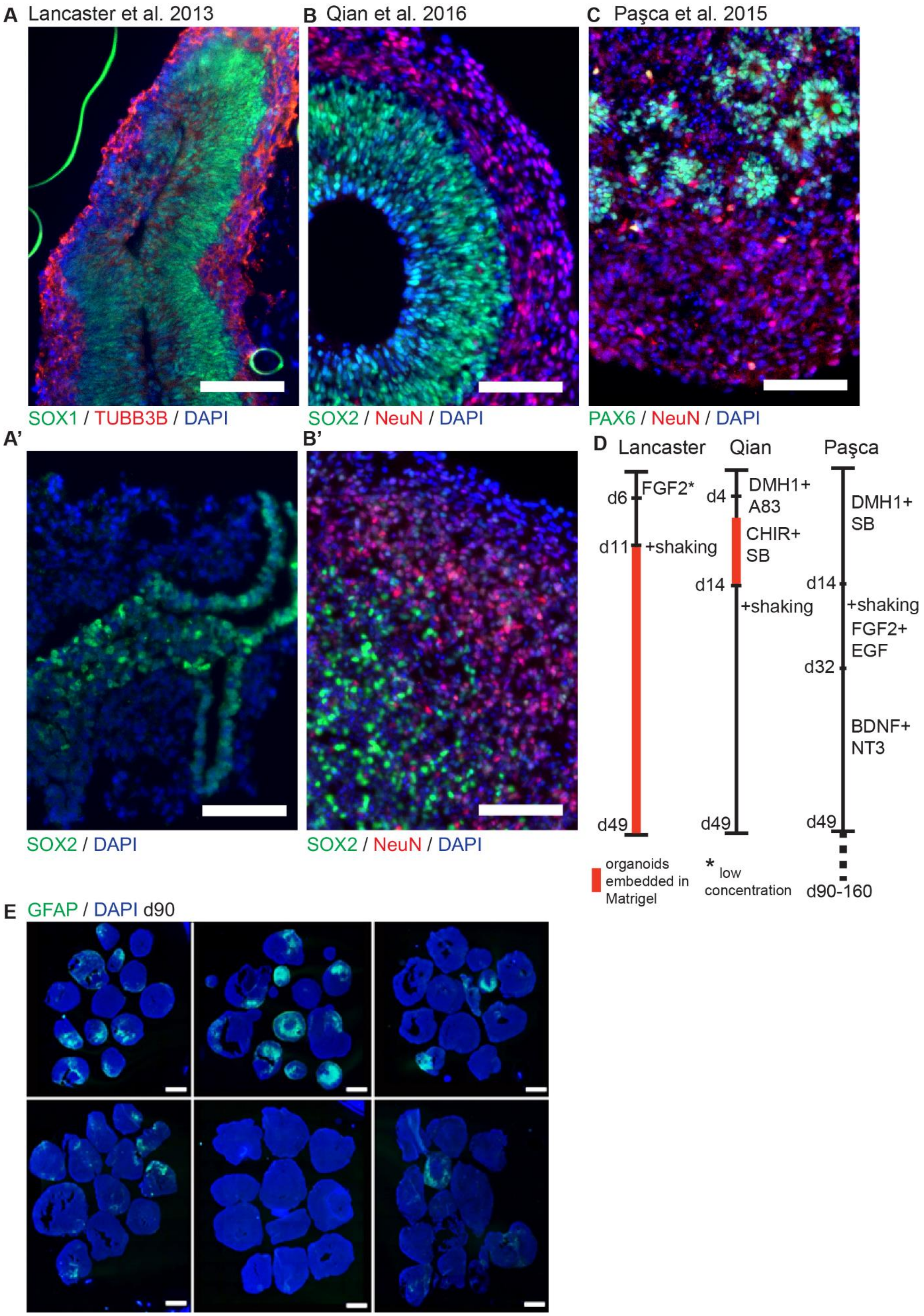

A Lancaster et al. 2013
B Qian et al. 2016
C Paşca et al. 2015
SOX1 / TUBB3B / DAPI
SOX2 / NeuN / DAPI
PAX6 / NeuN / DAPI
A'
B'
SOX2 / DAPI
SOX2 / NeuN / DAPI
D
Lancaster
Qian
Paşca
d6
FGF2*
d11
+shaking
d49
d4
DMH1+
A83
CHIR+
SB
d14
+shaking
d49
DMH1+
SB
d14
+shaking
FGF2+
EGF
d32
BDNF+
NT3
d49
d90-160
organoids embedded in Matrigel
* low concentration
E GFAP / DAPI d90

Differences in estimated cell type proportions in disomic and trisomic samples.

Table S1: pilot organoid study shown in Figure 2C. **Table S2**: expanded organoid study shown in Figure 4B. Columns are as follows: **cell.type**: cell types from Tanaka (2020) used as basis for deconvolution of bulk RNA-seq data; **ave.dis:** mean of estimated proportion of cell type in disomic samples; **ave.tris:** mean of estimated proportion of cell type in trisomic samples**; diff:** difference between these means (ave.dis - ave.tris); **df:** degrees-of-freedom for test of difference in proportion off cell type between disomic and trisomic samples using Welch unequal variance t-test; **p.value:** p-value for Welch t-test**; p.adj_bonf**: Bonferroni-corrected p-value, to control for multiple hypothesis testing (one test per cell-type).

Table S1. Pilot study organoid cell-type composition

Cell type	Ave dis	Ave tris	diff	df	P value	p.adj Bonferroni
Astrocyte	2.97e-01	3.80e-01	-8.27e-02	4.72	0.1640	1.000
BMP responsible cell	6.67e-19	6.94e-18	-6.27e-18	4.07	0.3542	1.000
Cilia bearing cell	2.90e-21	8.71e-03	-8.71e-03	4.00	0.1784	1.000
Cortical neuron	9.31e-02	3.05e-01	-2.12e-01	7.99	0.0291	0.378
Glia progenitor cell	1.08e-02	1.30e-01	-1.19e-01	4.45	0.0180	0.234
Intermediate	3.46e-18	2.91e-17	-2.57e-17	4.17	0.2239	1.000
Interneuron	4.92e-01	8.67e-02	4.05e-01	4.54	0.0414	0.538
Mesoderm	1.06e-18	2.61e-18	-1.55e-18	4.90	0.5957	1.000
Neuroepithelial cell	3.72e-02	1.45e-18	3.72e-02	4.00	0.0844	1.000
Neuron	2.35e-17	1.92e-17	4.31e-18	7.98	0.8699	1.000
Oligodendrocyte OPC	9.48e-08	1.40e-17	9.48e-08	4.00	0.3739	1.000
Proteoglycan expressing cell	1.64e-03	4.58e-18	1.64e-03	4.00	0.3739	1.000
Unfolded protein responsible cell	6.80e-02	8.98e-02	-2.18e-02	7.67	0.8096	1.000

Table S2. Expanded study organoid cell-type composition

Cell type	Ave dis	Ave tris	diff	df	P value	P adj Bonferroni
Astrocyte	9.01e-02	1.00e-01	-9.96e-03	2.86	0.836	1
BMP responsible cell	0.00e+00	2.42e-19	-2.42e-19	2.00	0.423	1
Cilia bearing cell	2.27e-06	3.64e-21	2.27e-06	2.00	0.423	1
Cortical neuron	2.05e-01	2.90e-01	-8.46e-02	4.00	0.636	1
Glia progenitor cell	1.82e-01	1.79e-01	3.33e-03	2.60	0.962	1
Intermediate	7.10e-18	3.95e-18	3.15e-18	3.71	0.638	1
Interneuron	3.66e-01	2.15e-01	1.51e-01	3.83	0.290	1
Mesoderm	5.66e-02	5.32e-02	3.46e-03	4.00	0.953	1
Neuroepithelial cell	4.90e-02	6.25e-02	-1.35e-02	2.33	0.646	1
Neuron	2.08e-02	5.63e-02	-3.54e-02	2.44	0.567	1
Oligodendrocyte OPC	2.86e-02	1.48e-02	1.39e-02	3.41	0.378	1
Proteoglycan expressing cell	1.35e-03	8.75e-03	-7.40e-03	2.37	0.229	1
Unfolded protein responsible cell	1.93e-21	2.08e-02	-2.08e-02	2.00	0.322	1

Supplemental Table S3: Results from tests of differential gene expression in organoid studies. **(Excel spreadsheet Table S3)**

Sheet 1 gives results without cell-type adjustment and **Sheet 2** gives results with adjustment for estimated proportion of cortical neurons, both for the comparions of three disomic vs three trisomic cell lines. **Sheet 3** gives results for the pilot study of 5 vs 5 organoids from a single disomic and trisomic cell line.

Columns in each sheet are as follows: **gene_id** is the Ensembl ID for the gene, and **gene_name** is the corresponding gene name from the Ensembl gtf (relase 87); **logFC** is the log2(fold-change) of expression levels in trisomic / disomic organoids; **logCPM** is the average log2(counts-per-million) across all samples; **PValue** is the P-value for differential expression; **FDR** is the FDR-adjusted P-Value using all genes; **FDR_chr21** and **FDR_non21** are FDR-adjusted P-values computed separately for genes on chr21 and genes not on chr21 (see discussion in Material and Methods for rationale). Genes are sorted by PValue.
